# Supplementary figures and images for: Bis-Indole Derivatives as Dual Nuclear Receptor 4A1 (NR4A1) and NR4A2 Ligands
Source: Biomolecules. 2024 Feb 27;14(3):284. doi: 10.3390/biom14030284 (PMC10967861; doi:10.3390/biom14030284)

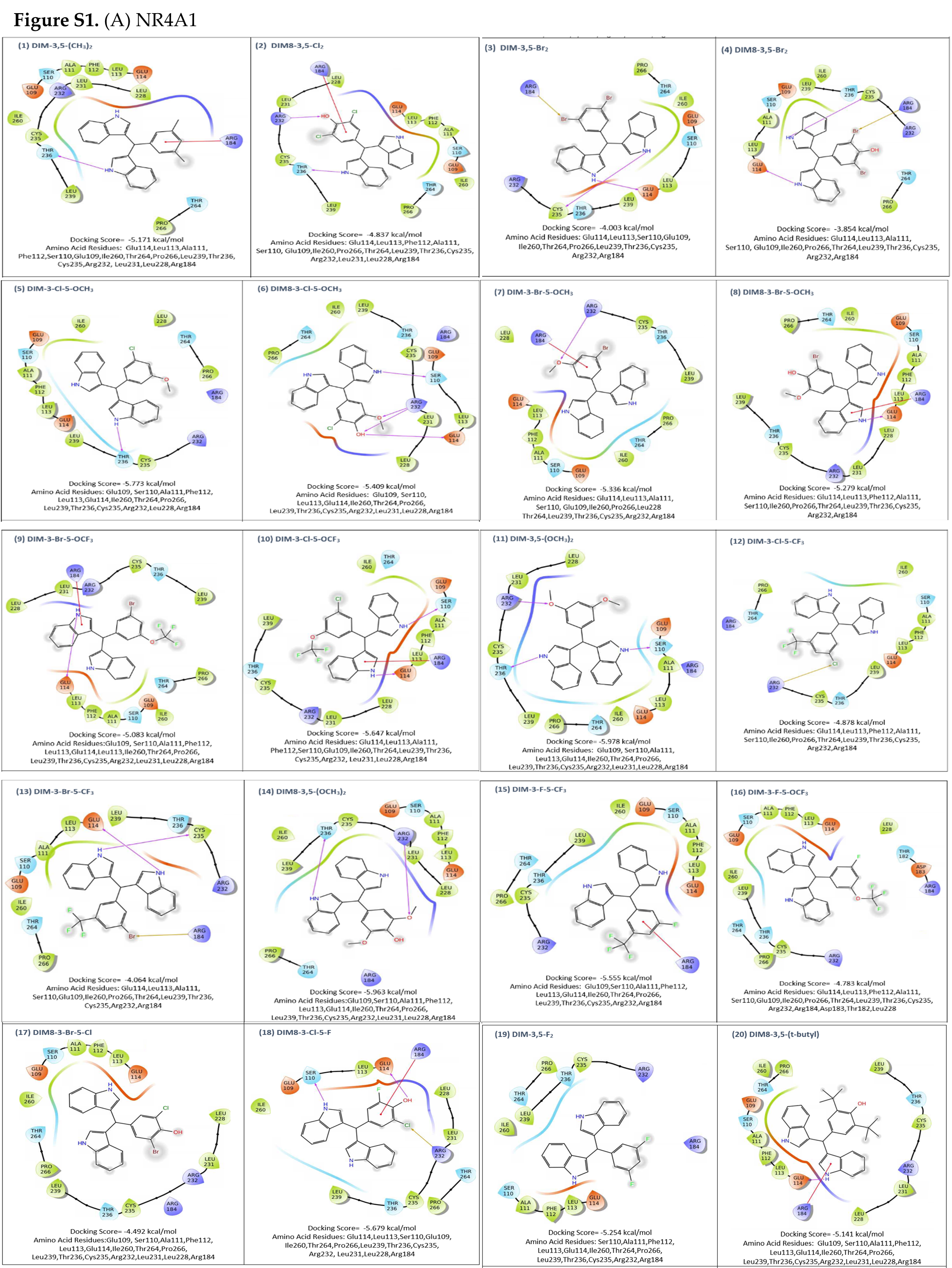

Supplement: Supplementary file 1 [file biomolecules-14-00284-s001.zip › S1 A NR4A1.png]

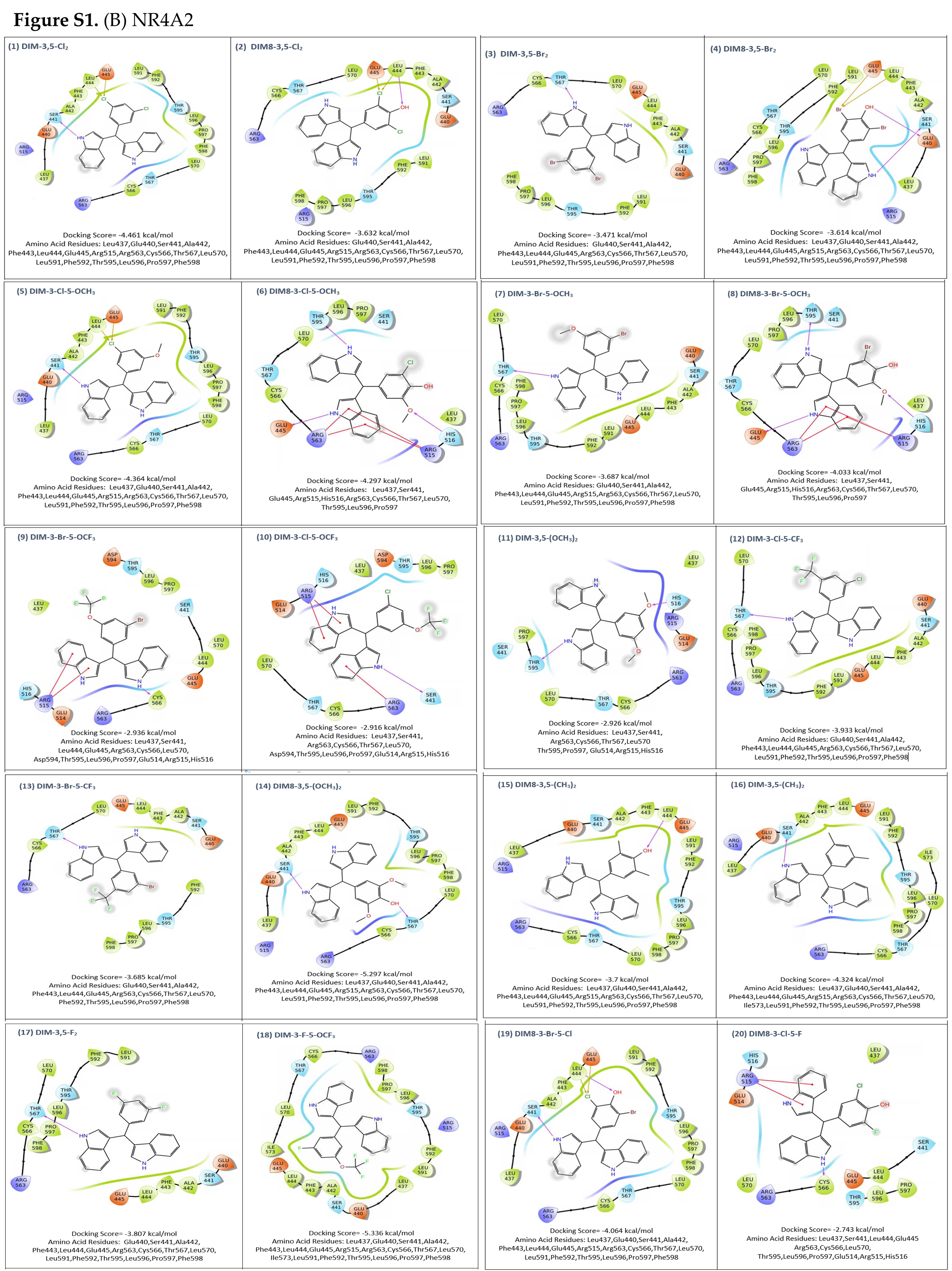

Supplement: Supplementary file 1 [file biomolecules-14-00284-s001.zip › S1 B NR4A2.png]
